# 3-dimensional versus conventional laparoscopy for benign hysterectomy: protocol for a randomized clinical trial

**DOI:** 10.1186/s12905-017-0434-7

**Published:** 2017-09-07

**Authors:** Elise Hoffmann, Gitte Bennich, Christian Rifbjerg Larsen, Jannie Lindschou, Janus Christian Jakobsen, Pernille Danneskiold Lassen

**Affiliations:** 1 Zealand University Hospital, Koegevej 7-13, 4000 Roskilde, Denmark; 2 Roskilde University Hospital, Koegevej 7-13, 4000 Roskilde, Denmark; 3 Herlev University Hospital, Herlev Ringvej 75, 2730 Herlev, Denmark; 4 Copenhagen Trial Unit, Rigshospitalet Copenhagen University Hospital, Tagensvej 22, 2200 Copenhagen, Denmark; 5 Department of Cardiology, Holbæk Hospital, Holbæk, Denmark

**Keywords:** Conventional laparoscopy, 3-dimensional laparoscopy, Hysterectomy, Daily living, Postoperative pain

## Abstract

**Background:**

Hysterectomy is one of the most common surgical procedures for women of reproductive age. Laparoscopy was introduced in the 1990es and is today one of the recommended routes of surgery. A recent observational study showed that operative time for hysterectomy was significantly lower for 3-dimensional compared to conventional laparoscopy. Complication rates were similar for the two groups. No other observational studies or randomized clinical trials have compared 3-dimensional to conventional laparoscopy in patients undergoing total hysterectomy for benign disease.

The objective of the study is to determine if 3D laparoscopy gives better quality of life, less postoperative pain, less per- and postoperative complications, shorter operative time, or a shorter stay in hospital and a faster return to work or normal life, compared to conventional laparoscopy for benign hysterectomy.

**Methods/design:**

The design is a randomised multicentre clinical trial. Participants will be 400 women referred for laparoscopic hysterectomy for benign indications. Patients will be randomized to 3-dimensional or conventional laparoscopic hysterectomy. Operative procedures will follow the same principles and the same standard whether the surgeon’s vision is 3-dimensional or conventional laparoscopy. Primary outcomes will be the impact of surgery on quality of life, assessed by the SF 36 questionnaire, and postoperative pain, assessed by a Visual Analogue scale for pain measurement. With a standard deviation of 12 points on SF 36 questionnaire, a risk of type I error of 3.3% and a risk of type II error of 10% a sample size of 190 patients in each arm of the trial is needed. Secondarily, we will investigate operative time, time to return to work, length of hospital stay, and - and postoperative complications.

**Discussion:**

This trial will be the first randomized clinical trial investigating the potential clinical benefits and harms of 3-dimensional compared to conventional laparoscopy. The results may provide more evidence regarding the future place of 3-dimensional laparoscopy in the range of endoscopic approaches for benign hysterectomy.

**Trial registration:**

This study is registered at ClinicalTrial.gov: NCT02610985 November 16th 2015. November 2015. The regional Ethical committee approved it on the 12. November 2015, approval number: SJ-498. Data handling was approved by the Danish Data Protection Agency: REG-109-2015 on the 13. November 2015.

## Background

Today it is generally accepted that the procedures of choice for the removal of uterus on benign indication should be laparoscopic or vaginal surgery. Both procedures are associated with shorter hospital stay and faster recovery compared to laparotomy [1–4]. Vaginal hysterectomy has lower costs and in some studies, a lower complication rate compared to laparoscopy [5]. In younger women without prolapse, however, the operation is not as straightforward as in older women. Furthermore, proper access to the pelvic cavity is not provided by the vaginal route, which makes laparoscopy first choice when the procedure involves adnexal surgery. In many departments, adnexal surgery has become part of the standard procedure for hysterectomy, since recent studies suggest that concomitant removal of the fallopian tubes may decrease the risk of development of ovarian and peritoneal cancer [6].

Despite promising results, adoption of the laparoscopic approach for benign hysterectomy has been slow. A longer operating procedure, the requirement of advanced laparoscopic skills, and challenges in especially obese patients have been cited as the main reasons [7]. Accordingly even today only a minor proportion of all benign hysterectomies are performed laparoscopically in European countries.

In 2005 the US Food and Drug Administration approved the Da Vinci robotic platform for gynaecologic surgery. Due to improved dexterity, ergonomics, and a 3-dimensional imaging the technology was promoted to overcome many of the limitations and drawbacks of conventional laparoscopy [8]. Within few years, the number of benign hysterectomies performed by robotic-assisted laparoscopy showed a tremendous rise. Benefits compared to conventional laparoscopy, however, have not been reported [9]. On the contrary, a randomized trial concludes that robotic assistance may lengthen operative time for benign hysterectomy with an average of 77 min compared to conventional laparoscopy [10].

Three-dimensional (3D) laparoscopy offers the same 3-dimensional stereoscopic imaging as robotic surgery, but in a conventional laparoscopic set-up with much lower costs [11]. Although dexterity and ergonomics are theoretically inferior to those of robotic surgery, docking of the big and inconvenient patient robotic-cart is avoided, reducing the number of operative steps. Furthermore, instruments are held by the surgeon and not by robotic arms, which means a preservation of the tactile sense. The place of 3D laparoscopy in the range of endoscopic approaches for benign hysterectomy, however, is not clear. An updated literature search in PubMed and Embase, using Mesh terms and the search string *““Imaging, Three-Dimensional”” [Mesh]) AND ((““Hysterectomy”” [Mesh]) OR laparoscopic hysterectomy) (19–02-2015)*, demonstrates that data are sparse. No randomized trials exist, and only a single retrospective study has been published within gynaecological surgery. In that study, operative time for hysterectomy was significantly lower for 3D compared to conventional laparoscopy. Complication rates were similar for the two groups [12].

In conclusion, although laparoscopy is one of the recommended routes of surgery for benign hysterectomy, the place of 3D laparoscopy in the range of endoscopic approaches is not clear. Robotic surgery for hysterectomy was introduced without evidence for the clinical benefit, underlining the importance of clinical trials before implementation of new techniques. We expect that 3D- technology improves imaging and facilitates faster and safer surgery, causing less pain, shorter recovery and fewer postoperative complications.

## Methods and design

### Aim

The objective of the study is to determine if 3D laparoscopy gives better quality of life, less postoperative pain, less per- and postoperative complications, shorter operative time, or a shorter stay in hospital and a faster return to work or normal life, compared to conventional laparoscopy for benign hysterectomy.

### Design

An investigator-initiated, blinded, randomized, clinical trial.

### Setting

Roskilde University Hospital, Denmark.

Herlev University Hospital, Denmark.

### Participants

**Fig. 1:**
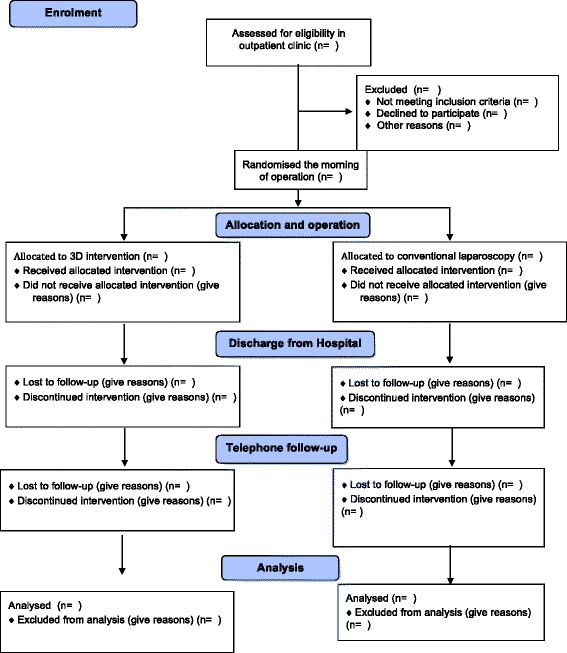
Participant flowchart in accordance with the CONSORT statement

Participants will be patients referred for laparoscopic hysterectomy with or without bilateral salpingectomy or bilateral salpingo-oophorectomy for benign indications. Women not suitable for laparoscopic hysterectomy, either because of [1] ultrasound appraisal of uterus weight > 1000 g or [2] decision to carry out concomitant prolapse surgery, will not be included. Further, patients not able to read and understand Danish or give informed consent are also excluded (Fig 1).

### Interventions

In order to assess which mode of laparoscopy is more favourable in a RCT, it is necessary to use similar and well-defined procedures in all other areas around the surgery. Therefore the preoperative information and patient education is standardized. Pre-emptive medication is 8 mg dexamethasone, 2 g paracetamol, 400 mg Ibumetin and 4 mg Ondacetron. Anaesthesia and postoperative care is also standardized as well as the operative procedure.

Standard preoperative assessment will be performed together with transvaginal ultrasound estimating the size of the uterus. Operative procedures will follow the previously described “Gold standard” as follows: A manipulator will be used in all patients to expose and delineate different anatomical structures. For ligation and dissection, a Thunderbeat® bipolar/ultrasound sealing and cutting energy device will be used. After detachment, uterus will be removed through the vaginal vault. In case of a very narrow vagina combined with a myomatous uterus it may be necessary to enlarge the abdominal incision [13]. Morcellation or any other destruction of the uterus inside the abdomen will not be performed. The vaginal vault will be closed laparoscopically with a running Vicryl 2–0 suture or a barbed suture V-lock® 2–0. Urinary catheter will be removed immediately after the operation. For postoperative pain relief, the patients will be given sufentanil/Fentanyl in the recovery ward, and paracetamol, NSAID and in some cases morphine in the gynaecological unit. After a few hours on the recovery ward, they will return to the gynaecological unit.

### Data collection

Patients found suitable for participation will be informed about the trial. If the patient wants to participate she will fill out a quality of life questionnaire (SF 36-v2).

*If willing to participate, and after instructions on how to understand the questionnaire the patient completes the SF-36 questionnaire in the preoperative assessment. To reduce risk of bias at the 6 week postoperative telephone interview, patient is re-introduced to and provided with a copy of the SF 36 questionnaire at the time of discharge; further, the research nurse is instructed to stay neutral at the telephone interview* Randomization will be performed in the morning of the operation day. A visual analogue-scale (VAS) for pain scoring will be used when the participants return to the gynaecological unit, 5 h postoperatively. Hospital discharge will be considered when the women are fully mobilized, urinating and pain is controlled with milder oral analgesia. At discharge, use of painkillers stronger than over-the-counter medicine will be registered. The women will be asked to scale their pain at a VAS score in the morning of the first 3 days after discharge. Six weeks after surgery, all patients will receive a telephone call from a nurse, where a SF-36-v2 questionnaire similar to that given preoperatively will be filled out. Pre-operative data is registered by the doctor in the outpatient department record sheet, Operative data by the surgeon in the operation record sheet, and Postoperative data recorded by the nurse surgeon in the discharge department record sheet. Any readmission or complications causing readmission is registered by the doctor in the readmission sheet and will be double-cheeked at the telephone interview. If readmitted or treated at another hospital, this will be registered (linked to personal identification number) at the national patient register where all diagnoses are registered and compiled, with the Hysterectomy Database ensuring no complication will be overlooked.

Time to return to work or other normal life, use of postoperative painkillers, and postoperative complications are noted by the nurse in the telephone interview 6 weeks after the operation (Table 1).

**Table 1:** Plan for obtaining data for both 2- and 3D hysterectomies

3D and conventional laparoscopic hysterectomy	Enrolment in outpatient clinic	Allocation and operation	Discharge from hospital	Telephone interview
TIMEPOINT	-t1	0	tx	tx + 6 weeks
Baseline variables: height, weight, age, prior operations, chronic pain, parity, estimated weight of uterus, indication for operation, site. Quality of life by SF-36 questionnaire	x	x		
Outcome variables 1: operating time, bleeding, concomitant surgery, weight of uterus		x		
Outcome variables 2: Postoperative pain and medication	x		x	
Outcome variables 3: re-admissions, complications at home, return to work, pain, return to daily living, unexpected malignancy. Quality of life by SF-36 questionnaire	x			x

### Randomization

Randomization will be performed centrally by The Copenhagen Trial Unit. The allocation sequence will be computer-generated and hidden from the investigators and participants. The allocation ratio will be 1:1 and stratification variables will be parity (0 or more), centers (Roskilde or Herlev) and previous larger pelvic surgery (yes/no).

### Blinding

The surgeon operating the patient cannot, for obvious reasons, be blinded. All patients and all caregivers (except the surgeons) at the clinical departments will all be blinded to trial intervention allocation. This is possible because patches on the stomach are placed the same way regardless of conventional or 3D surgery. The medical doctors evaluating the patient condition after surgery and discharging the patients will also be blinded to the trial intervention allocation.

### Outcomes

#### Primary outcomes

1. The impact of surgery on quality of life, assessed by questionnaire (SF-36-v2, physical health score) at a telephone interview 6 weeks postoperatively.
2. *Postoperative pain is assessed on a 100 mm VAS-scale immediately after the operation at the admission to post anaesthesia care unit (PACU) and again 5 h postoperatively at gynaecological unit or at the PACU if the patient unexpectedly has not returned to gynaecological unit. These measures are related to the patient lying quiet in the bed. Further pain related to physical movements, will be reported in the morning on day 1–3 postoperatively (longitudinal data). Postoperative pain will also be related to the analgesics given as we register the use of units of morphine during the first 3 days.*

#### Secondary outcomes

1. Proportion of participants with one or more of the following major complications: organ lesion (injury to bowel, bladder or ureter), vaginal hematoma requiring intravenous AB, vaginal cuff rupture, port hernia, reoperation due to bleeding, deep vein thrombosis, pulmonary emboli or severe cardiac complications and mechanic ileus. *Conversion to laparotomy is categorized as a major complication if done for any other reason than to remove a large uterus. In cases of conversion (mini-laparotomy) to remove a large uterus, mini-laparotomy is not considered a complication, and these patients are pre-operatively informed of the risk of this event.*
2. Proportion of participants with one or more of the following of minor complications: Cystitis, smaller vaginal cuff hematomas without need for intravenous antibiotics, port infection orpain with normal findings by vaginal ultrasound. All major and minor adverse events will also be assessed separately.
3. The impact of surgery on quality of life, assessed by questionnaire (SF-36-v2, mental health score).
4. Length of hospital stay (hours).
5. Operative time (defined as time from placement of manipulator until last suture).

When calling the participants 6 weeks after their operation, we will also ask the women if and when they returned to work and these reports will be summarized for exploratory purposes.

### Statistical analyses

The statistical analysis plan will be described in detail and published in “BMC Medical Research Methodology”.

Our primary analyses for all outcomes will be adjusted for the three stratification variables (number of previous births (0 or more), centres (Roskilde or Herlev), and previous larger pelvic surgery (yes/no)). Our secondary analysis for all outcomes will be adjusted for the three stratification variables (see above) and other prognostic variables (history of chronic pelvic pain (yes/no), co-morbidity, enlarging of the abdominal incision to remove uterus (yes/no), multiple adherences or need for other laparoscopic procedures (yes/no). Full analysis will be carried out on both groups, the intention-to-treat population (all randomized participants) and observed cases.

Longitudinal data will be analysed using area under the curve, continuous data will be analysed linear regression, dichotomous data will be analysed using logistic regression, and survival data will be analysed using cox regression. We will perform two sided statistical tests.

We will assess if the thresholds for statistical significance and clinical significance are crossed using the five-point procedure as suggested by Jakobsen et al. [14]. This procedure will include adjustments of thresholds for significance according to the number of primary outcomes (*P*-value threshold 0.033) and number of randomized participants. We will use a Bayes factor threshold for significance of 0.1 based on the prior anticipated intervention effect (see sample size calculation).

We will register if postoperative pathologic examination unexpectedly reveals cancer in a stage indicating further surgery, or if surgery must be converted to open surgery, for other reasons than to remove the uterus. However, we expect such cases to be rare and we will therefore include such participants in the primary analysis. Sensitivity analyses will be performed if the proportion of such cases is larger than expected.

#### Interim analyses and early stopping

After collection of data from 100 patients, an independent safety and data monitoring committee will perform the first interim analysis. This committee will thereafter advise the steering committee and have the responsibility of monitoring whether the trial should be stopped early or continued until the sample size has been reached.

### Sample size calculation and power estimation

Because our trial is designed with two primary outcomes, our sample size calculation will be based on a type 1 error of 3.3% [15].

#### Postoperative pain

We use a 100 mm VAS-score to assess pain. We consider 10 mm as the minimal relevant difference with a standard deviation of 20 mm and we accept a risk of type I error of 3,3% and a risk of type II error of 10%. This results in a sample size of 92 patients in each arm of the trial.

#### SF 36 quality of life

We consider 4 points on the SF 36 quality of life as the minimal relevant difference with a standard deviation of 12 points and we accept a risk of type I error of 3.3% and a risk of type II error of 10%. This results in a sample size of 190 patients in each arm of the trial.

To be able to assess both of the co-primary outcomes, we therefore chose 2 × 190 participants as our necessary sample size.

### Power estimations for secondary outcomes based on a sample size of 2 × 190 participants

#### Major and minor complications

With a 5% acceptable risk of type I error, a proportion of major complications in the control group of 12%, a relative risk reduction of 20%, we will have 8.7% power when assessing this outcome.

#### Length of hospital stay (hours)

With a sample size of 190 women in each arm of the study, an alpha value of 5% and an average discharge of 48 h postoperatively versus 40 h, and a standard deviation of 25 h, we have a power of 84%.

#### Operative time

With a sample size of 190 women in each arm of the study, an alpha value of 5% and an average operation time of 101 min in the 2D group, and an operation time of 90 min in the 3D group, and a standard deviation of 36 min, we have a power of 81%.

### Missing data

All analyses are performed according to the intention- to-treat principle. In case of less than 5% of missing data for the randomized participants for primary and secondary outcome, a complete case analysis will be performed. In case of more than 5% missing data, multiple imputations will be used to handle missing data. We will additionally perform a sensitivity analysis using a best-worst and worst-case scenario imputation [14].

#### Ethical considerations

When conducting medical intervention studies, ethical considerations are always essential. This trial is important because it is in the interest of the patient as well as the health system to optimise surgery and minimise adverse outcomes without an unreasonable increase in medical device expenses. Gynaecological examination and operation are associated with discomfort to some extent, but patients with relevant symptoms are undergoing these procedures whether or not they are included in the trial. Furthermore, available evidence suggests that 3D technology is at least as good as the current technology. Thus, we provide the best-proven standard of operation for both groups, and it is very unlikely that we increase risks for the patients by performing 3D laparoscopy. Further all relevant information including undesirable medical events occurring during the clinical trial, presumably caused by the operation, will be reported to the Danish Hysterectomy and Hysteroscopy Database (DHHD).

We have designed the trial so that the risk of systematic errors and bias is minimised and therefore, data will be meaningful and improve future laparoscopic surgery.

## Discussion

The presented randomized trial is designed to investigate the beneficial and harmful effects of 3-dimensional versus conventional laparoscopy in patients undergoing benign laparoscopic hysterectomy. A previous retrospective study suggests that operative time for hysterectomy is significantly lower for 3D compared to conventional laparoscopy [12]. Complication rates were not statistically different, but this conclusion was limited by a small number of patients in each group. So far, no trials have examined whether 3D laparoscopy has an impact on postoperative pain or quality of life after return to daily activities.

3D laparoscopy offers a 3-dimensional stereoscopic imaging combined with preserved tactile sense as in a conventional laparoscopic set-up, and 3D laparoscopy might therefore refine and optimise surgery. However, we do not know whether 3D laparoscopy offers clinical benefit compared to conventional laparoscopic, whether the potential beneficial effects are relevant for the patient, or whether 3D laparoscopy causes unexpected adverse effects.

The price of 3D laparoscopy is higher than for 2D equipment, but lower than for robotic surgery. As medical equipment is becoming increasingly sophisticated and expensive, it is essential to determine whether 3D has relevant effects in clinical practise before it is implemented as part of the daily routine.

We hope that this trial will help place 3D laparoscopy in the range of endoscopic approaches for benign hysterectomy.

### Trial strengths and limitations

This trial has a number of strengths. First, the randomized design makes it possible to validly assess the effects of the two surgical interventions. Second, the trial has a high degree of external validity because we assess the effects of operative procedures as they are performed in most gynecological departments and we use two recruiting centers. Third, the risk of systematic errors is reduced by central randomization stratified for prognostic factors [16, 17], blinding is used to the highest possible extent, and data is analysed according to the intention-to-treat principle. Fourth, in trials investigating operative procedures it is often difficult to distinguish whether different effects of the operative techniques or varying skills of the surgeon cause a supposed difference. In this present trial the same experienced surgeons perform both the conventional and 3D operations. This, together with the overall low risk of bias of this trial, contributes to a high degree of internal validity. Finally, we estimated a sample size adjusted for two primary outcomes using realistic anticipated intervention effects and we performed power calculations for all secondary outcomes, and we planned to adjust the thresholds for significance if we do not reach the planned sample size. This limits the risks of random error [14].

A limitation of this trial is that we will probably not be able to detect a difference in complication rate between the 2 arms of the trial. The overall low complication rate following laparoscopic hysterectomy requires a large sample size to detect a possible difference. We expect it is more likely to demonstrate a difference in postoperative pain or quality of life, two other relevant clinical outcomes that have not previously been assessed following 3D laparoscopic hysterectomy.

## Abbreviations

3D: 3-dimensional
Tv. UL: Transvaginal ultrasound
UL: Ultrasound
